# Bowel perforation surgery due to fecal impaction immediately after hernia mesh surgery (TAPP)

**DOI:** 10.1093/jscr/rjac583

**Published:** 2022-12-17

**Authors:** Jung Bum Choi, Byoung Chul Lee, Jeong Hee Han, Byeong Gwan Noh, Jae Kyun Park, Young Mok Park, Hyuk Jae Jung, Hong Jae Jo

**Affiliations:** Department of Surgery, Pusan National University Hospital, 179, Gudeok-ro, Seo-gu, Busan 49241, Korea; Department of Surgery, Pusan National University Hospital, 179, Gudeok-ro, Seo-gu, Busan 49241, Korea; Department of Surgery, Pusan National University Hospital, 179, Gudeok-ro, Seo-gu, Busan 49241, Korea; Department of Surgery, Pusan National University Hospital, 179, Gudeok-ro, Seo-gu, Busan 49241, Korea; Department of Surgery, Pusan National University Hospital, 179, Gudeok-ro, Seo-gu, Busan 49241, Korea; Department of Surgery, Pusan National University Hospital, 179, Gudeok-ro, Seo-gu, Busan 49241, Korea; Department of Surgery, Pusan National University Hospital, 179, Gudeok-ro, Seo-gu, Busan 49241, Korea; Department of Surgery, Pusan National University Hospital, 179, Gudeok-ro, Seo-gu, Busan 49241, Korea

## Abstract

Inguinal hernia repair using prosthetic mesh is used as a standard treatment in most countries and considered superior to primary suture repair. Although prosthetic mesh has greatly reduced the risk of recurrence, the risk of mesh infection remains. A 71-year-old man was diagnosed with symptomatic bilateral inguinal hernias. He underwent successful laparoscopic transabdominal preperitoneal (TAPP) repair and was discharged the same day. After 3 days, he was diagnosed with small bowel perforation, and underwent emergency surgery. We found perforation of the distal ileum caused by the fecal impaction and severe intra-abdominal contamination. We performed subtotal colectomy and ileosigmoid anastomosis, but did not remove the prosthetic mesh because the previous TAPP site was intact. The patient recovered well post-operatively. Therefore, contaminated or dirty surgery immediately after the hernia mesh surgery could be a feasible treatment.

## INTRODUCTION

Transabdominal preperitoneal hernia (TAPP) repair is an approved and common surgical approach for the treatment of inguinal hernia in adults, particularly for bilateral and recurrent inguinal hernia after open surgery [1]. With the advent of prosthesis, the outcome of hernia repairs has indeed improved significantly. But there are still other issues to be resolved, including mesh infection. In the case of ventral hernia repair using mesh, it is known that the use of prosthetic mesh in infected fields should be prohibited, and has been reported that superficial infections occur 2.5 and 3.8 times more frequently in cleanliness-contaminated and contaminated patients, respectively, than in cleanliness patients [2]. It is crucial that the use of prosthetic mesh in contaminated hernias should be restricted regardless of the level of contamination. However, there is no consensus on the use of prostheses in potentially infected surgical fields in cases of TAPP and there is no specific guideline when the mesh is contaminated shortly after hernia repair. We would like to share our experience of a patient who had perforated peritonitis 3 days following TAPP surgery.

## CASE REPORT

**Figure 1:**
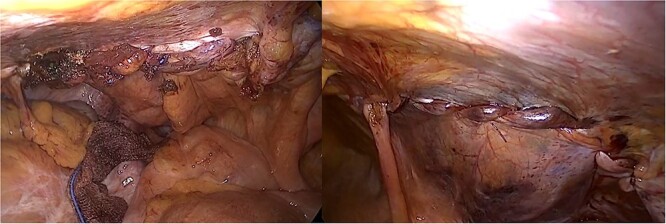
Laparoscopic images after laparoscopic hernia repair.

A 71-year-old man was diagnosed to the surgical department with symptomatic bilateral inguinal hernias and he had undergone robot-assisted radical prostatectomy 2 years ago. The bilateral inguinal hernias were evident on clinical. He underwent successful laparoscopic TAPP repair and was discharged on the operation day (Fig. 1).

**Figure 2:**
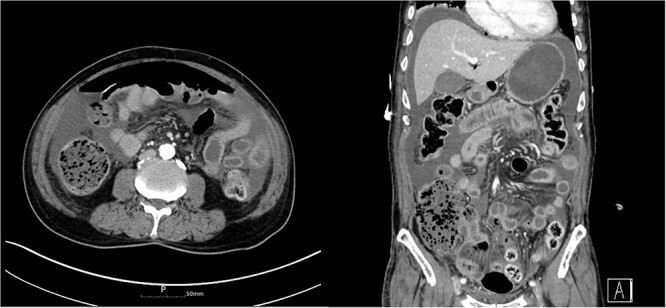
Abdomen CT showed complicated fluid collection and free air in the abdominal cavity.

Three days after discharge, he presented to the emergency room with severe abdominal pain. He did not have bowel movement for a week. He had severe constipation and frequently dug up the stool with his fingers. A physical examination revealed abdominal distension and rigidity with rebound tenderness. The patient’s blood pressure was 110/70 mmHg, pulse rate was 116 beats/min and body temperature was 38.0°C. In the complete blood count, a white blood cell count was 2.04 × 10^3^/mm^3^ and hemoglobin was 13.5 g/dL. The C-reactive protein was 34.33 mg/dL, procalcitonin was 14.50 ng/mL and creatinine was 1.79 mg/dL. He underwent computed tomography (CT), which showed focal bowel wall defect at the distal ileum, and complicated fluid collection and free air in the abdominal cavity (Fig. 2). The patient was diagnosed with small bowel perforation due to fecal impaction and underwent emergency surgery.

**Figure 3:**
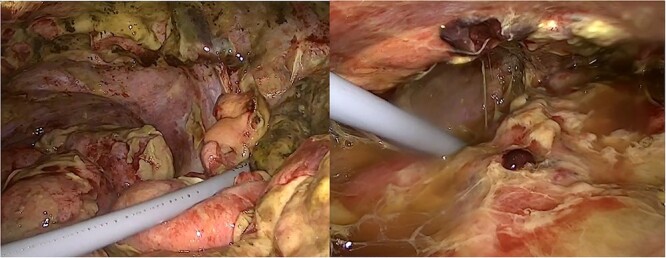
Laparoscopic images of bowel perforation and severe intra-abdominal contamination.

On laparoscopic exploration, we found perforation of the distal ileum caused by the fecal impaction and severe intra-abdominal contamination (Fig. 3). The entire colon was filled with very hard stool and the cecum was dilated up to 11 cm. We decided to perform surgery for bowel perforation and constipation at the same time. We performed subtotal colectomy and ileosigmoid anastomosis, but did not remove the prosthetic mesh because the previous TAPP site was intact. The patient recovered well and was discharged on post-operative day 9. He has been followed up during the last 1 year and shown no signs of infection, and he has a normal stool once a day.

## DISCUSSION

Numerous studies revealed that patients who underwent laparoscopic repair had low recurrence rate, had fewer post-operative complications, were discharged earlier and were able to return to their daily lives more quickly than patients who underwent open hernia [2]. However, they did not clarify the best laparoscopic approach for inguinal hernia repair. TAPP is becoming popular in herniorrhaphy, especially when previous herniorrhaphy is open or both hernia operations are required. Various complications have been reported, such as pain, infection, recurrence and so on. Among them, the frequency of mesh infection is very low (0.1–0.2%) [3].

Belansky *et al.* [4] describe the pathophysiology of mesh infection, which is contaminated by bacteria within the first 24–48 h and is not integrated with surrounding tissues, forming an impermeable membrane, such as biofilm, without blood flow. Once a biofilm is formed on the surface of the mesh, infection cannot be removed, and the treatment is often difficult. So, in most cases, even in those where antibacterial agents are preserved and cured, recurrence is repeated, and the mesh is finally removed. Therefore, many surgeons are paying attention to intra-operative infections.

Contamination of a field during a hernia repair can occur due to many factors such as an inadvertent enterotomy or need for concomitant bowel resection at the time of repair [2]. Xourafas *et al.* [5] examined the impact of mesh on ventral hernia repair with a simultaneous bowel resection and found a significantly higher incidence of infections and other complications in patients that had mesh versus patients without mesh. It is recommended that the safe method in laparoscopic hernia corrections with unintended contamination of the gastrointestinal tract, reproductive urology or biliary tract is not to use the artificial mesh at the same time as restoring organ damage, but instead to redesign fundamental hernia corrections within weeks [2].

On the other side, it has not been demonstrated that there is an increased risk of mesh contamination in the event of simultaneous operations performed on the digestive tract. Some authors report prosthetic repair of the abdominal wall after colonic resection with good results [6, 7]. Many others perform prosthetic inguinal hernia repair in emergencies that require intestinal resection in cases of strangulated hernias [1]. It is also common for laparoscopic surgeons to perform inguinal prosthetic hernia repair TAPP after performing a cholecystectomy [8].

Regarding TAPP surgery, there were some cases of bowel perforation and the timing of occurrence was different for all reported cases, but mesh site infection was not observed during the follow-up [9]. In our case, intra-abdominal contamination occurred only 3 days after surgery, but no signs of infection were observed during the follow-up period for the patient, and there were no abnormal findings on the CT. We assume that placing the mesh in the preperitonal space leads to the isolation of the peritoneal cavity from the mesh with less risk of contamination [10]. Contaminated or dirty surgery immediately after hernia mesh surgery could be a feasible treatment.
